# Cystic Neutrophilic Granulomatous Mastitis: A Case Series

**DOI:** 10.7759/cureus.113531

**Published:** 2026-07-28

**Authors:** Visalini Sridharan, Moses Ambroise, Oshan Saini, Desdemona Rasitha, Sheela Devi Chandrakesan

**Affiliations:** 1 Pathology, Pondicherry Institute of Medical Sciences, Puducherry, IND; 2 Pathology, Histopathology, Pondicherry Institute of Medical Sciences, Puducherry, IND; 3 Microbiology, Pondicherry Institute of Medical Sciences, Puducherry, IND; 4 Microbiology, Pondicherry Institute Of Medical Sciences, Puducherry, IND

**Keywords:** corynebacterium species, cystic neutrophilic granulomatous mastitis, granulomatous mastitis, lipid vacuoles, lobulocentric granulomatous inflammation

## Abstract

Cystic neutrophilic granulomatous mastitis (CNGM) is a distinct histopathological subtype of granulomatous mastitis that commonly presents as an inflammatory breast lesion in women of reproductive age. We present a retrospective case series of five parous women who presented with unilateral breast lumps between 2023 and 2025. Histopathological examination revealed classical features of CNGM in all cases. Microbiological culture yielded various isolates, including *Corynebacterium tuberculostearicum*, *Tsukamurella* species, and *Mycolicibacterium fortuitum *complex, while two cases showed no growth. The findings highlight a heterogeneous microbial spectrum in CNGM and emphasize the importance of microbiological evaluation for accurate diagnosis and appropriate antimicrobial therapy.

## Introduction

Cystic neutrophilic granulomatous mastitis (CNGM) is a distinct subtype of granulomatous lobular mastitis owing to its unique histopathological features. The histological pattern is characterized by a central lipid vacuole surrounded by neutrophils and an outer layer of epithelioid histiocytes, multinucleated giant cells, and lymphocytes. Occasionally, Gram-positive, rod-shaped bacilli can be seen inside the lipid vacuoles. It is often associated with *Corynebacterium* species [1,2].

In 2003, Taylor et al. published a historic series of 34 breast abscesses with unique histologic characteristics linked to *Corynebacterium* infection. The majority of them were granulomatous, and all of them had characteristic vacuoles with suppurative inflammation. These vacuoles were assumed to reflect dissolved lipid. Gram staining identified bacilli in 16 cases [3]. This entity was subsequently recognized as cystic neutrophilic granulomatous mastitis, characterized by distinctive histopathologic features and a strong association with *Corynebacterium*, as demonstrated in studies by Renshaw et al. and D. Alfonso et al. [4,5].

CNGM is a rare histopathological diagnosis, constituting less than 1% of breast specimens examined histologically. Clinically, CNGM typically presents as a unilateral breast mass in women of reproductive age, often following a history of breastfeeding. Although it typically presents as a unilateral lesion, bilateral involvement has been reported in approximately 8.5% of patients with CNGM [4]. Even though the exact pathogenesis of CNGM remains unclear, lactation-related changes are believed to play an important role. Retained milk secretions due to impaired drainage may result in ductal dilatation and epithelial damage, leading to leakage of lipid-rich material into the surrounding tissue and subsequent granulomatous inflammation. In addition, secondary colonization by *Corynebacterium* species and other microorganisms has been implicated in disease pathogenesis. Proposed risk factors include pregnancy, breastfeeding, hyperprolactinemia, endocrine disorders, and breast trauma [5].

Clinically, CNGM commonly presents as a breast mass and may be associated with nipple inversion, sinus formation, pain, nipple discharge, erythema, or abscess formation. Its overlapping clinical and radiological features with breast carcinoma and infective mastitis often pose diagnostic challenges, necessitating histopathological confirmation [4].

Given the rarity of CNGM, its ability to clinically and radiologically mimic breast carcinoma and other infective breast lesions, and the growing recognition of diverse microbial associations, we report a series of five cases highlighting the importance of histopathological examination and microbiological evaluation in establishing an accurate diagnosis.

This retrospective case series includes 5 female patients aged 24-49 years who were evaluated between 2023 and 2025 and initially presented with clinical provisional diagnoses of mastitis, breast abscess, or suspected malignancy. Clinical data, ultrasound imaging (BI-RADS (Breast Imaging Reporting and Data System) classification), and fine-needle aspiration cytology (FNAC) were reviewed. Histopathology included Hematoxylin and Eosin (H&E) stain, periodic acid-Schiff (PAS) stain, Grocott-Gomori’s methenamine silver (GMS) stain, Ziehl-Neelsen (ZN) for acid-fast bacilli, and Gram staining. Microbiological workup was done for all cases.

## Case presentation

Case 1

**Figure 1:**
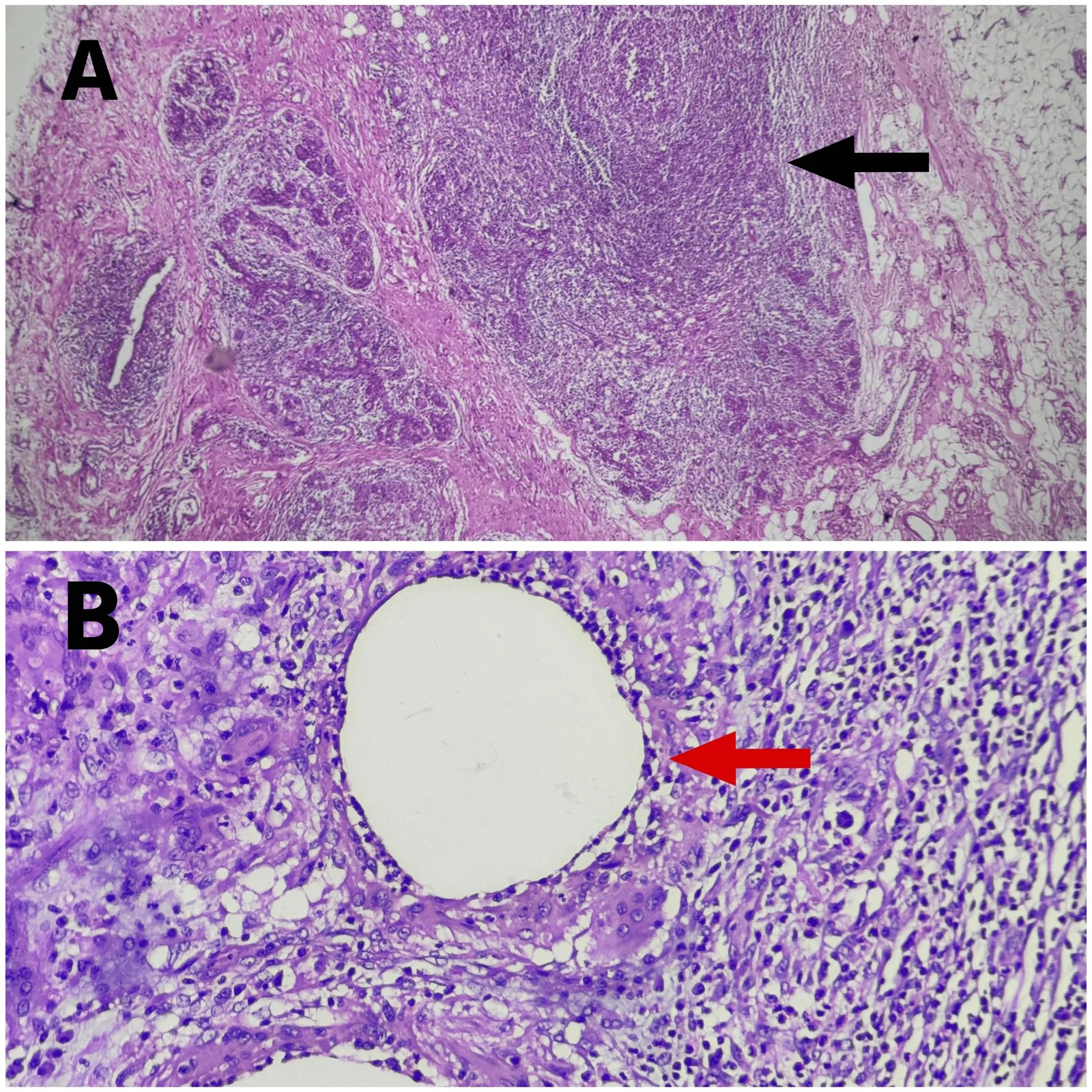
Histopathological images A: H&E stain showing a lobulocentric granulomatous inflammation (black arrow) (40x). B: H&E stain showing a central lipid vacuole surrounded by neutrophils, epithelioid cells, Langhans giant cells, and lymphocytes (red arrow) (400x). (H&E: Hematoxylin and Eosin)

**Figure 2:**
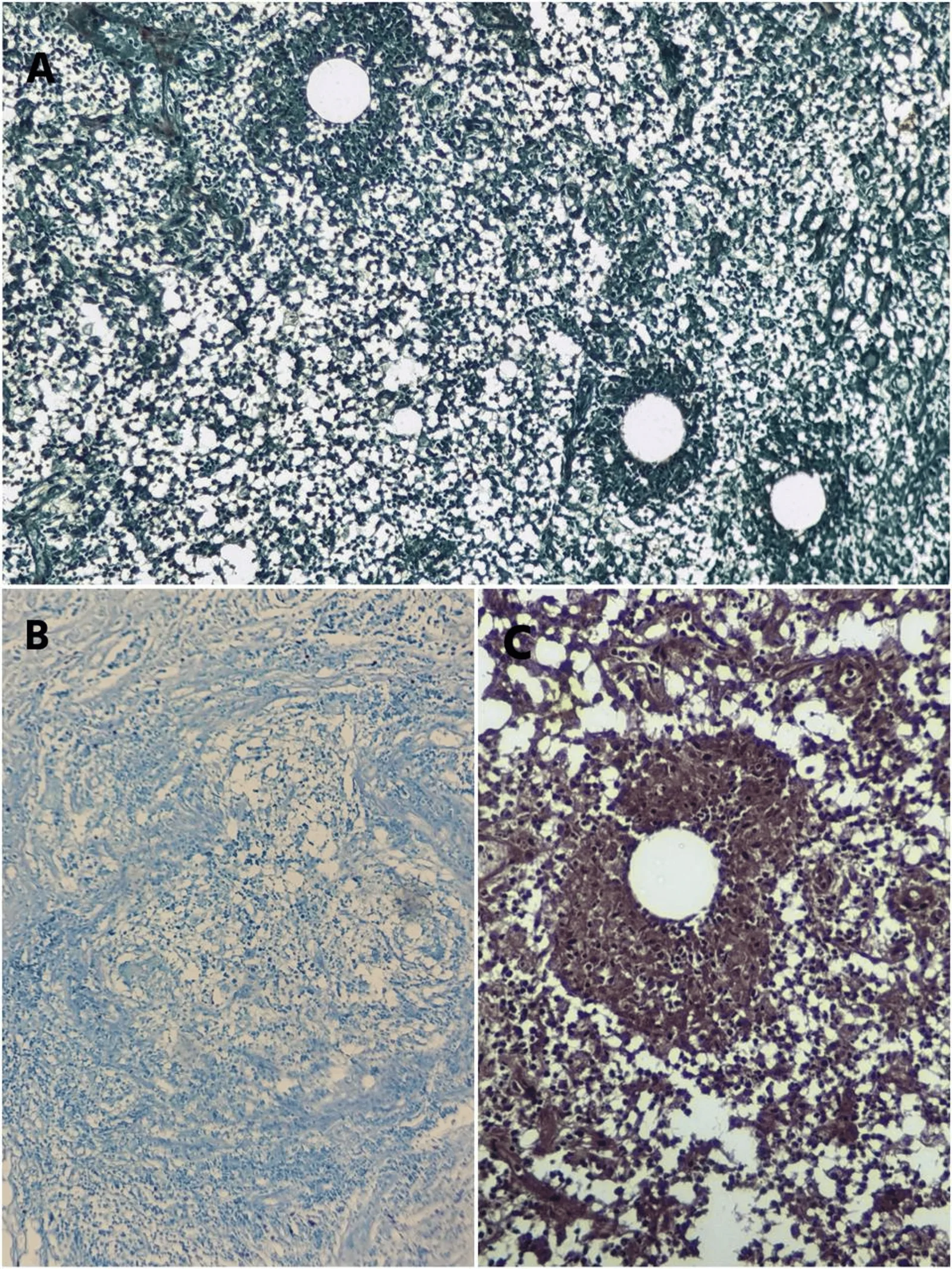
Special stains A: GMS stain: Negative for fungal elements; B: ZN stain: Negative for acid-fast bacilli; C: Gram stain: Negative for bacilli in lipid vacuoles (GMS: Grocott-Gomori’s Methenamine Silver, ZN: Ziehl-Neelsen)

**Figure 3:**
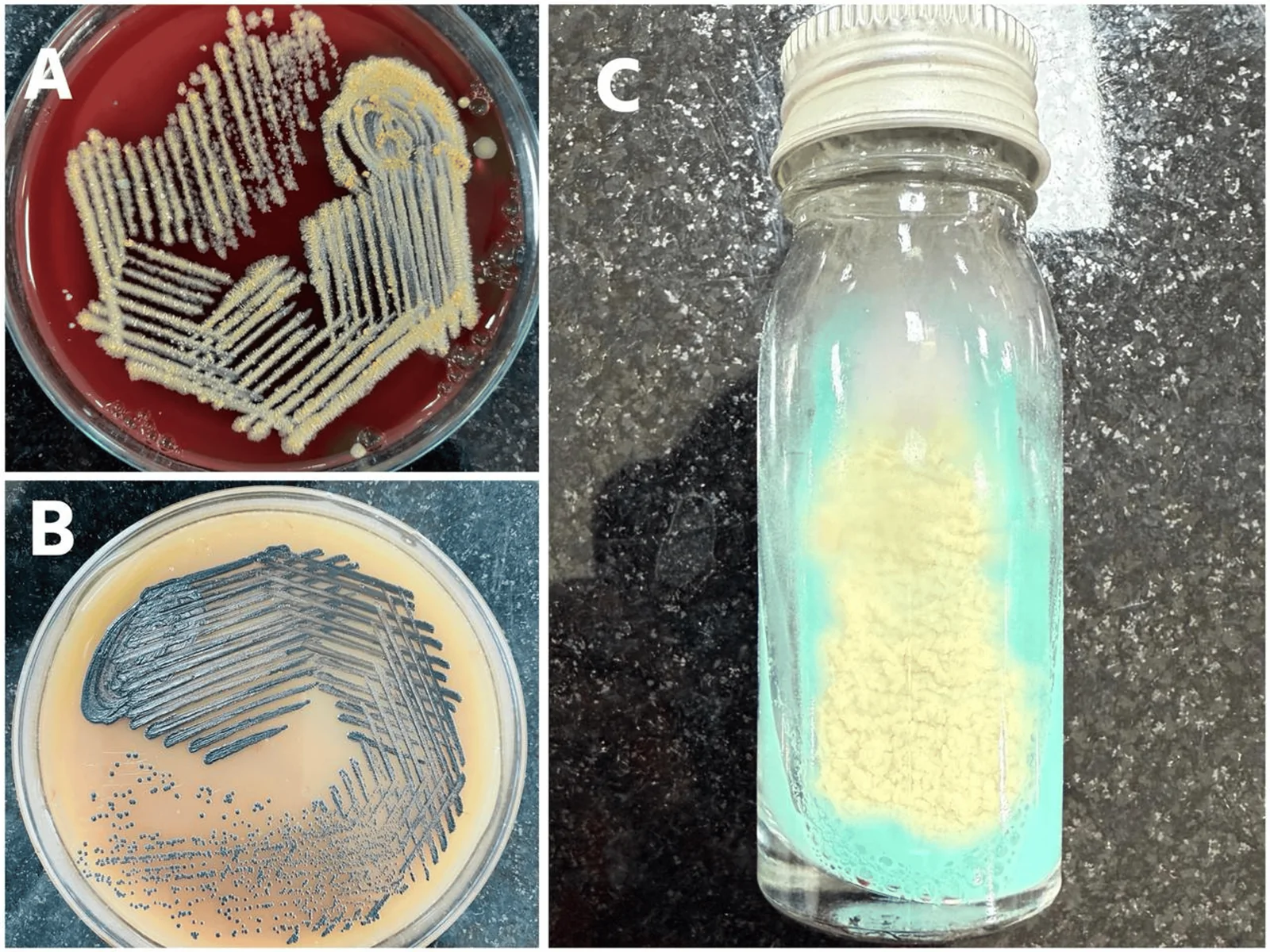
Culture images A: *Tsukamurella* species: Blood agar shows dry, yellow, non-hemolytic colonies on prolonged incubation. B: *Corynebacterium tuberculostearicum*: Black-colored colonies on potassium tellurite agar. C: *Mycolicibacterium fortuitum*: Smooth, moist cream- to buff-colored colonies on Lowenstein-Jensen medium.

A 28-year-old female presented with left breast swelling associated with pain for two weeks. There was no history of fever, nipple discharge, or trauma before she visited the hospital. She had associated endocrine disorders, namely, type 2 diabetes mellitus and hypothyroidism. She was para one, living one (P1L1), with a history of breastfeeding. On examination, a lump measuring 15.0 × 8.0 cm was found involving the central, upper, and outer quadrants of the left breast. The swelling was firm in consistency and freely mobile. Based on the clinical findings, a provisional diagnosis of mastitis was made. Ultrasound revealed features of acute mastitis of the left breast. FNAC yielded thick, yellowish purulent material. Cytology showed numerous neutrophils and macrophages, with few epithelioid granulomas and multinucleated giant cells suggestive of granulomatous suppurative mastitis. As symptoms persisted, an incision and drainage procedure was performed. The tissue submitted for histopathology revealed clear lipid vacuoles rimmed by neutrophils with an outer cuff of epithelioid histiocytes and Langhans-type giant cells, forming the characteristic pattern of CNGM (Figures 1A, 1B). GMS stain was negative for fungal elements, and ZN stain for acid-fast bacilli was also negative. Gram stain did not reveal any organism inside the lipid vacuole (Figures 2A-2C). Microbiological culture from aspirated fluid showed growth of *Tsukamurella* species (Figure 3A). Matrix-assisted laser desorption/ionization time-of-flight mass spectrometry (MALDI-TOF) confirmed the isolate as *Tsukamurella*, strengthening the microbiological results. The patient was treated with linezolid following microbiological identification of *Tsukamurella* species. She remained asymptomatic during follow-up, with no evidence of recurrence over six months.

Case 2

**Figure 4:**
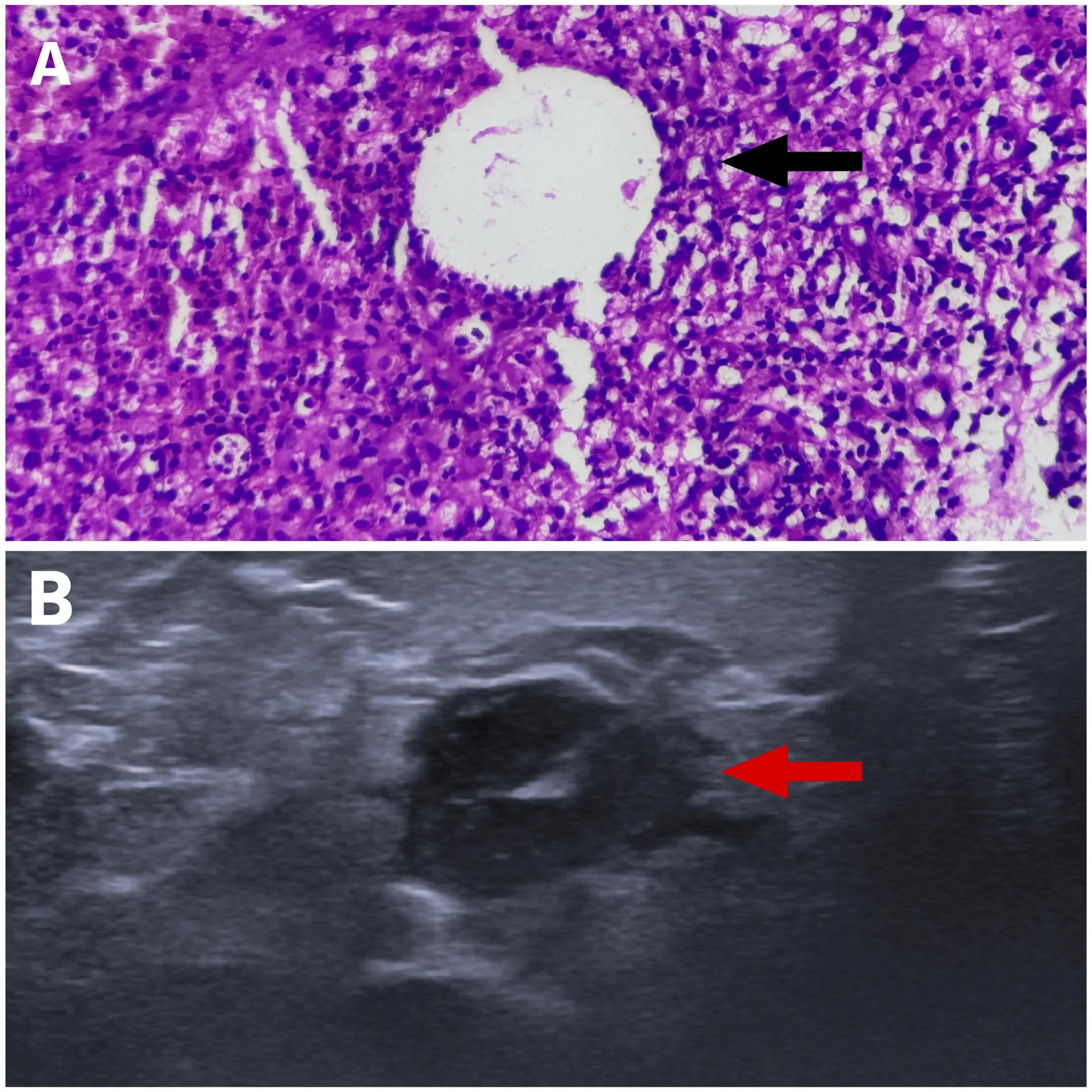
Histopathology and radiology images A: H&E stain showing a central lipid vacuole surrounded by neutrophils, epithelioid cells, Langhans giant cells, and lymphocytes (black arrow) (400x). B: Ultrasonogram of a breast abscess showing well-defined hypoechoic collections with mobile internal echoes (red arrow). (H&E: Hematoxylin and Eosin)

A 49-year-old female presented with pain and swelling in the left breast for two months, associated with pus discharge and intermittent low-grade fever. There was no history of trauma. She was a known case of hypothyroidism with no other known co-morbidities. She was para two, living two (P2L2), with a history of breastfeeding. On clinical examination, an approximately 15.0 × 10.5 cm cystic-to-firm swelling was noted involving the medial half of the left breast, extending beyond the nipple-areolar complex (NAC) into the lateral quadrants. Two ulcers were identified: one measuring 0.5 × 0.5 cm at the six o’clock position, located 0.5 cm from the NAC, and another measuring 1 × 0.5 cm at the nine o’clock position, located 1 cm from the NAC. Associated nipple retraction and peau d’ orange changes of the overlying skin were also present. Based on the clinical findings, a provisional diagnosis of malignancy was considered. Ultrasonography revealed a relatively well-defined hypoechoic collection in the left breast, reported as a likely evolving abscess (BI-RADS 3) (Figure 4B). Fine-needle aspiration cytology showed features of fat necrosis. Subsequently, a trucut biopsy was performed, which demonstrated the characteristic histopathological features of CNGM (Figure 4A). Special stains were negative for fungal organisms and acid-fast bacilli. Microbiological culture of the pus yielded *Corynebacterium tuberculostearicum *(Figure 3B). The patient was treated with amoxicillin-clavulanic acid and remained free of recurrence during follow-up for six months.

Case 3

**Figure 5:**
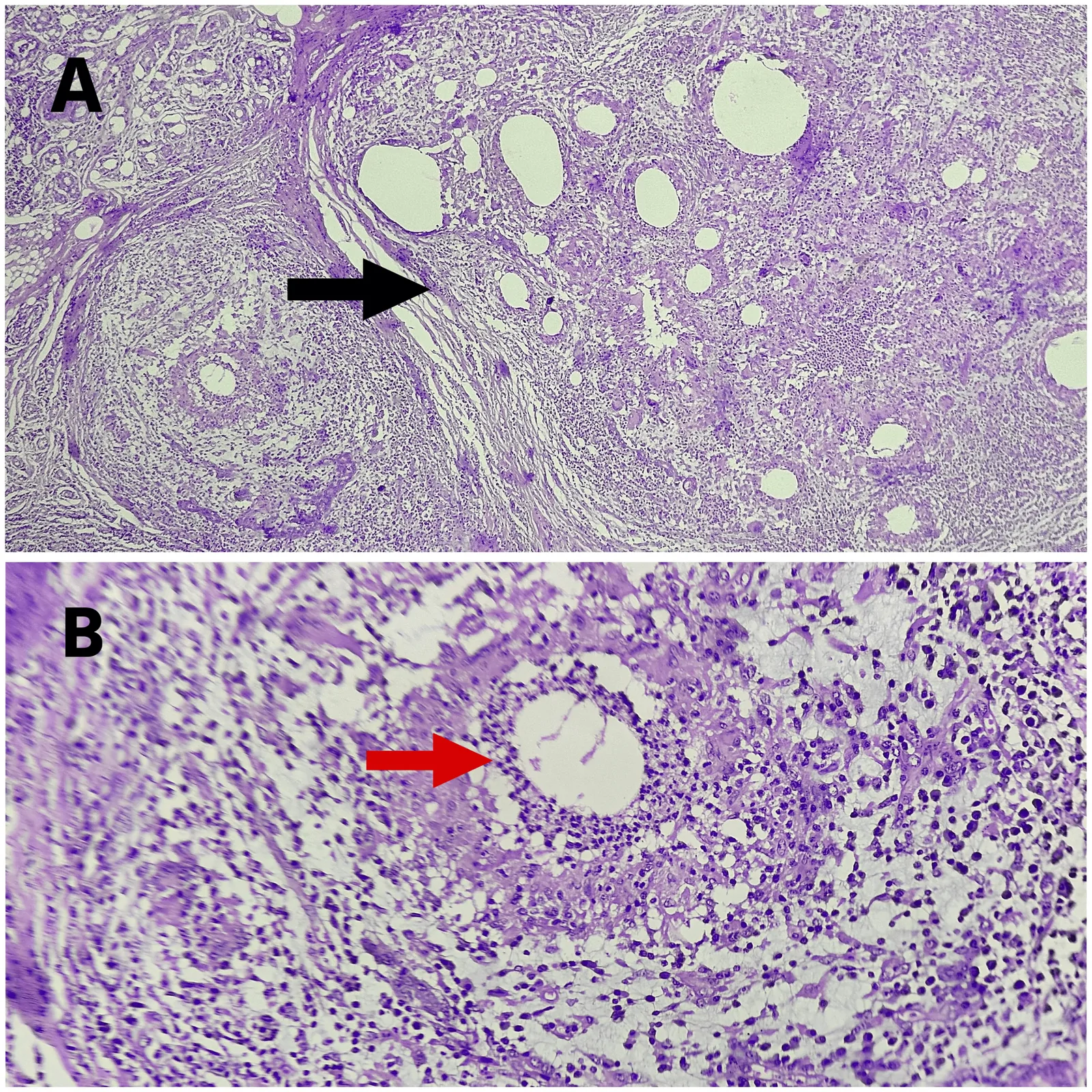
Histopathology image A: H&E stain showing a lobulo-centric granulomatous inflammation (black arrow) (x40). B: H&E stain showing a lipid vacuole rimmed by neutrophils and surrounded by lymphocytes and epithelioid cells (red arrow) (x200). (H&E: Hematoxylin and Eosin)

A 27-year-old female presented with a 3-month history of a left breast lump associated with pain and nipple retraction. There was no history of pus discharge, fever, or trauma. She had a recently diagnosed endocrine disorder, type 2 diabetes mellitus. She was para two, living two (P2L2), with a history of breastfeeding. On clinical examination, two lumps were identified in the left breast. The larger lump measured 6.0 × 5.0 cm and extended from the 11 o’clock to one o’clock position, while the smaller lump measured 4.0 × 3.0 cm and extended from the three o’clock to four o’clock position. The provisional clinical diagnosis was a breast abscess. Ultrasonography also revealed two lesions. The lesion at the three o’clock position appeared as a hypo- to isoechoic solid lesion, suggestive of an organized abscess or antibioma (BI-RADS 3). The lesion at the 12 o’clock position appeared as a thick-walled cystic lesion, along with a few dilated thick-walled lactiferous ducts showing surrounding inflammatory changes (BI-RADS 3). The lesion at the three o’clock position was excised and submitted for histopathological examination, which demonstrated the characteristic features of CNGM (Figure 5). Special stains were negative for fungal organisms and acid-fast bacilli. Microbiological culture of the pus showed no growth. The patient received cefixime as medical management. Recurrence was noted after six months of follow-up. Further management with incision and drainage in conjunction with antibiotic therapy was advised; however, the patient elected to continue treatment elsewhere.

Case 4

**Figure 6:**
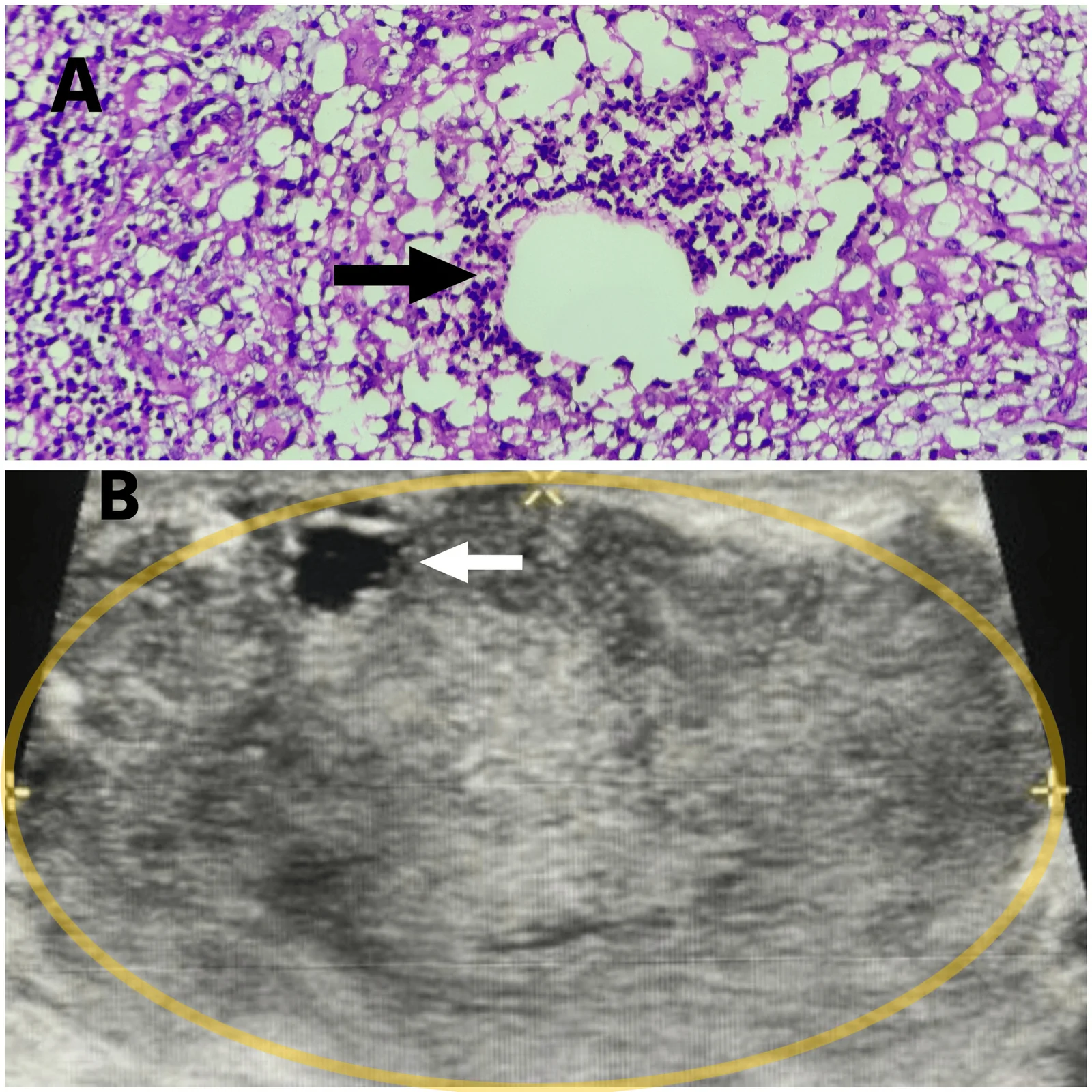
Histopathology and radiology image A: H&E stain showing a lipid vacuole rimmed by neutrophils and surrounded by lymphocytes, epithelioid cells (black arrow) (x200). B: Ultrasonogram of breast abscess showing a well-defined hypoechoic to isoechoic, predominantly solid lesion with tightly packed internal echoes in the center (yellow circle) and areas of cystic degeneration (white arrow).

A 35-year-old female presented with a left breast lump associated with pain and pus discharge from the nipple for a duration of three months. There was no history of fever or trauma. She had no associated endocrine disorders. She was para 1, living 1 (P1L1), with a history of breastfeeding. On clinical examination, a 5.0 × 4.0 cm lump was noted at the three-four o’clock position, with associated nipple retraction. The provisional clinical diagnosis was a breast abscess. Ultrasonography revealed two lesions at the three-four o’clock and one o’clock positions, appearing as ill-defined hypoechoic collections near the nipple-areolar complex, with surrounding diffuse inflammatory changes - likely a breast abscess (Figure 6B). A wide local excision was performed, and the specimen was submitted for histopathological examination, which demonstrated the characteristic features of CNGM (Figure 6A). Special stains were negative for fungal organisms and acid-fast bacilli. Microbiological culture of the aspirated fluid showed no growth. The patient was treated with linezolid and clindamycin and remained free of recurrence during follow-up for six months.

Case 5

**Figure 7:**
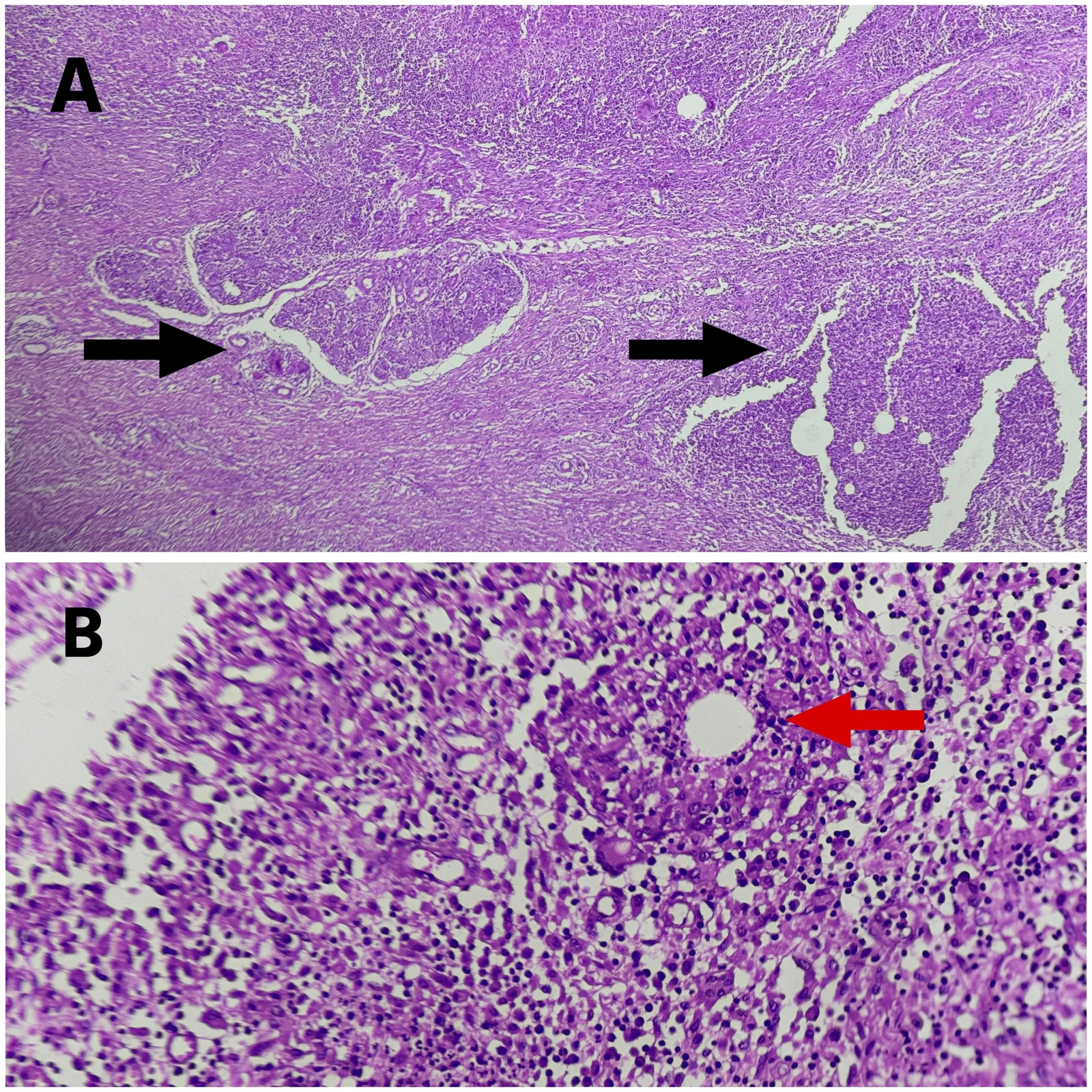
Histopathology image A: H&E stain showing lobulo-centric granulomatous inflammation (black arrows) (x40). B: H&E stain showing a lipid vacuole rimmed by neutrophils and surrounded by lymphocytes and epithelioid cells (red arrow) (x200). (H&E: Hematoxylin and Eosin)

A 24-year-old antenatal female at 15 weeks of gestation with no known co-morbidities presented with a two-month history of swelling in the right breast. There was no associated pain, nipple discharge, or fever. She had no associated endocrine disorders. She was gravida 2, para 1, living 1 (G2P1L1), with a history of breastfeeding. On clinical examination, the provisional diagnosis was mastitis. Ultrasonography revealed an ill-defined heterogeneously hypoechoic area at the three-to-six o’clock position with surrounding inflammatory changes, suggestive of a breast abscess (BI-RADS 3). Fine-needle aspiration cytology showed features of granulomatous mastitis. Histopathological examination demonstrated the characteristic features of cystic neutrophilic granulomatous mastitis (Figure 7). Special stains were negative for fungal organisms and acid-fast bacilli. Microbiological culture of the aspirated fluid yielded *Mycolicibacterium fortuitum *(Figure 3C). The patient was treated with imipenem, cotrimoxazole, and ciprofloxacin. Recurrence was noted after three months of follow-up, for which incision and drainage, along with antibiotic therapy, were advised; however, the patient declined further treatment.

The clinical, radiological, histopathological, and microbiological details are summarized in Table 1.

**Table 1:** Case details *During follow-up, incision and drainage, along with antibiotic therapy, were advised. However, the patients elected to pursue treatment elsewhere. (FNAC: fine-needle aspiration cytology; MALDI-TOF: matrix-assisted laser desorption/ionization time-of-flight mass spectrometry; CNGM: cystic neutrophilic granulomatous mastitis)

Case	Age (years)	Parity	History of breastfeeding	Co-morbidities	Clinical/Provisional diagnosis	Radiology findings	FNAC	Final Pathological diagnosis	Culture studies	Medical management	Follow-up	Follow-up duration
1	28	P1L1	Present	Type 2 diabetes mellitus, hypothyroidism	Mastitis	Acute mastitis	Not done	CNGM	Tsukamurella species (MALDI-TOF)	Linezolid	No Recurrence	6 months
2	49	P2L2	Present	Hypothyroidism	To rule out malignancy	Likely evolving abscess	Fat necrosis	CNGM	Corynebacterium tuberculostearicum (Figure 3B)	Amoxicillin, Clavulanic acid	No Recurrence	6 months
3	27	P2L2	Present	Recently diagnosed Type 2 diabetes mellitus	?Abscess	Likely organised abscess or antibioma	Not done	CNGM	No growth	Cefixime	Recurrence after 6 months*	6 months
4	35	P1L1	Present	Nil	Breast abscess	Likely abscess	Not done	CNGM	No growth	Linezolid, Clindamycin	No Recurrence	6 months
5	24	G2P1L1	Present	Nil	Mastitis	Likely breast abscess	Granulomatous mastitis	CNGM	Mycolicibacterium fortuitum (Figure 3C)	Imipenem, Cotrimoxazole, Ciprofloxacin	Recurrence after 3 months*	6 months

## Discussion

In this retrospective case series, five female patients who presented with a unilateral breast lump and pain were evaluated for suspected inflammatory breast disease. The presenting age group was of reproductive age, except case 2, who was in the perimenopausal age group. However, all of them had a history of childbirth and breastfeeding. Breastfeeding has been proposed as an important predisposing factor in the development of CNGM. Retained milk secretions and impaired drainage may induce a chronic inflammatory response within the breast, and the resulting ductal changes may create a favorable environment for bacterial colonization. These mechanisms may explain the higher frequency of CNGM among parous women with a history of breastfeeding, as observed in our series [3,5]. In our series, all patients were parous women with a history of breastfeeding, consistent with previous reports that have identified these as common clinical characteristics of CNGM.

In our series, breast masses and pain constituted the most common presenting symptoms, whereas ulceration and nipple discharge were less frequent findings. Comparable clinical presentations have been described by Wang et al. in studies of 62 and 141 cases of CNGM, where breast masses, pain, nipple discharge, and ulceration were among the commonly reported findings [5,6].

Except for cases 4 and 5, the rest of the patients had underlying endocrine disorders, such as type 2 diabetes mellitus or hypothyroidism, suggesting a possible association with CNGM. A study of 12 cases also highlighted endocrine disorders, apart from hyperprolactinemia, as potential risk factors for this infection [7].

The histopathological differential diagnosis of CNGM encompasses a broad spectrum of infectious and non-infectious granulomatous breast lesions. Non-infectious entities such as fat necrosis and silicone-induced granulomas may show lipogranulomatous inflammation; however, they lack the characteristic neutrophil-rimmed lipid vacuoles of CNGM, and silicone granulomas often contain polarizable foreign material within multinucleated giant cells. Autoimmune disorders involving the breast, including granulomatosis with polyangiitis and rheumatoid arthritis, should also be considered. Granulomatosis with polyangiitis is distinguished by necrotizing vasculitis, whereas rheumatoid nodules characteristically demonstrate central fibrinoid necrosis surrounded by palisading histiocytes and inflammatory cells without the formation of lipogranulomas. Ancillary investigations such as antineutrophil cytoplasmic antibody (ANCA) and rheumatoid factor testing may further support these diagnoses [8].

Breast carcinoma is also an important differential diagnosis of CNGM because both entities may present with a palpable breast mass, pain, and nipple inversion. Histopathological examination is therefore essential for establishing the diagnosis and excluding breast carcinoma. In the present series, one patient was initially evaluated with a provisional diagnosis to rule out malignancy before histopathological examination established the diagnosis of CNGM. Among infectious conditions, tuberculous mastitis is an important differential diagnosis, particularly in endemic regions. Unlike CNGM, tuberculosis typically exhibits well-formed caseating granulomas involving both ducts and lobules, with Ziehl-Neelsen staining, mycobacterial culture, and molecular assays, such as polymerase chain reaction (PCR), aiding confirmation. Sarcoidosis, although an uncommon cause of breast granulomatous inflammation, is characterized by compact, non-caseating granulomas composed predominantly of epithelioid histiocytes and multinucleated giant cells, without the neutrophilic microcysts that are characteristic of CNGM.The characteristic finding of lipid vacuoles rimmed by neutrophils with an outer cuff of epithelioid histiocytes and multinucleated giant cells, supported by negative special stains for fungal organisms and acid-fast bacilli and correlation with microbiological findings, is essential for excluding these differential diagnoses, avoiding diagnostic pitfalls and facilitating appropriate patient management [4]. However, early lesions lack the characteristic cystic lipid vacuoles rimmed by neutrophils and instead show a predominantly palisading granulomatous pattern. Such cases may represent an early stage in disease evolution. Although Gram staining is useful for demonstrating Gram-positive bacilli, false-negative results may occur because of the sparse distribution of organisms. Careful examination of the lipid vacuoles (microcysts) can improve the detection of these organisms [8].

*Corynebacterium* species are frequently associated with CNGM; however, other organisms, such as *Mycobacteroides* species, have also been reported [9], and a next-generation sequencing-based study by Wang et al. has further identified additional organisms, including *Pseudomonas aeruginosa*, implicating a broader microbial spectrum in disease pathogenesis [10]. Hence, a definitive etiological role remains unestablished. Our case series similarly demonstrated organisms beyond *Corynebacterium*, supporting a heterogeneous microbial spectrum. In our case series, two cases recurred.

Accurate identification of pathogens can be achieved using MALDI-TOF mass spectrometry for rapid species-level diagnosis in routine clinical settings. Molecular techniques, such as 16S rRNA and rpoB gene sequence amplification by PCR, can also be used [4].

Management of CNGM remains challenging because no standardized treatment protocol has been established, and recurrence has been reported despite various medical and surgical treatment strategies. In our series, all patients received antibiotic therapy, with surgical intervention performed when clinically indicated. Three patients remained recurrence-free during follow-up, whereas recurrence occurred in two patients. Given the small sample size and the observational nature of this case series, no conclusions regarding treatment efficacy or factors associated with recurrence can be drawn. Nevertheless, our findings are consistent with previous reports describing the variable clinical course, with persistence or recurrence reported in some patients despite treatment [4,8].

## Conclusions

CNGM is a distinct clinicopathological entity that has been reported predominantly in parous women of reproductive age with a history of breastfeeding. In the present series, all patients were parous women with a history of breastfeeding who presented with unilateral breast lesions that were initially diagnosed as mastitis, breast abscess, or suspected malignancy, reflecting the variable clinical presentation of this uncommon entity. The overlapping clinical and radiological features of CNGM with inflammatory and infective breast lesions contribute to its diagnostic difficulty.

Our case series highlights the heterogeneous microbiological spectrum associated with CNGM, including not only *Corynebacterium* species but also less commonly reported organisms such as *Tsukamurella* species and *Mycolicibacterium fortuitum* complex. The absence of bacterial growth in some cases may reflect limitations of culture techniques, prior antimicrobial therapy, or potentially heterogeneous disease mechanisms. These observations illustrate that microbiological findings in CNGM are variable and should be interpreted cautiously in conjunction with the clinical and pathological setting. In the present series, accurate diagnosis was achieved through correlation of the clinical presentation, radiological findings, histopathological examination, and microbiological evaluation. Such an integrated diagnostic approach is important for the recognition of this uncommon entity.
